# Population Genomics and Genetic Diversity of *Prosopis cineraria* in the United Arab Emirates: Insights for Conservation in Arid Ecosystems

**DOI:** 10.3390/plants14192970

**Published:** 2025-09-25

**Authors:** Anestis Gkanogiannis, Salama Rashed Almansoori, Maher Kabshawi, Mohammad Shahid, Saif Almansoori, Hifzur Rahman, Augusto Becerra Lopez-Lavalle

**Affiliations:** 1 International Center for Biosaline Agriculture (ICBA), Academic City, Al Ruwayyah 2, Dubai, P.O. Box 14660, United Arab Emirates; m.shahid@biosaline.org.ae (M.S.); r.hifzurrahman@biosaline.org.ae (H.R.); a.becerra@cgiar.org (A.B.L.-L.); 2 Environment Agency—Abu Dhabi (EAD), Al Mamoura Building (A), Muroor Road, Abu Dhabi, P.O. Box 45553, United Arab Emirates; mkabshawi@ead.gov.ae (M.K.); saifk.almansoori@ead.gov.ae (S.A.)

**Keywords:** *Prosopis cineraria*, Ghaf tree, population genomics, conservation genetics, genetic structure and diversity, desert biodiversity and restoration, United Arab Emirates

## Abstract

*Prosopis cineraria* (L.) Druce is a keystone tree species in the arid and semi-arid regions of West and South Asia, with critical ecological, cultural, and conservation significance. In the United Arab Emirates (UAE) and other regions of the Arabian Peninsula, this beneficial tree is called Ghaf. Despite its importance, genomic resources and population-level diversity data for the tree remain limited. Here, we present the first comprehensive population genomics study of Ghaf based on whole-genome resequencing of 204 individual trees collected across the UAE. Following Single-Nucleotide Polymorphism (SNP) discovery and stringent filtering, we analyzed 57,183 high-quality LD-pruned SNPs to assess population structure, diversity, and gene flow. Principal component analysis (PCA), sparse non-negative matrix factorization (sNMF), and discriminant analysis of principal components (DAPC) revealed four well-defined genetic clusters, broadly corresponding to geographic origins. The genetic diversity varied significantly among the groups, with observed heterozygosity (Ho), inbreeding coefficients (F), and nucleotide diversity (π) showing strong population-specific trends. Genome-wide fixation index F_ST_ scans identified multiple highly differentiated genomic regions, enriched for genes involved in stress response, transport, and signaling. Functional enrichment using Gene Ontology (GO), Kyoto Encyclopedia of Genes and Genomes (KEGG), and Pfam annotations indicated overrepresentation of protein kinase activity, ATP binding, and hormone signaling pathways. TreeMix analysis revealed gene flow into one of the genetic clusters from both others, suggesting historical admixture and geographic connectivity. This work provides foundational insights into the population genomic profile of *P. cineraria*, supporting conservation planning, restoration strategies, and long-term genetic monitoring in arid ecosystems.

## 1. Introduction

*Prosopis cineraria* (L.) Druce, locally known as the Ghaf tree, is a symbolic desert legume of the Arabian Peninsula. Recognized for its exceptional drought tolerance and ecological significance, this species functions as a keystone organism within arid ecosystems and is officially designated as the national tree of the United Arab Emirates (UAE). Its deep taproot system enables access to subterranean water sources, allowing it to maintain physiological activity during prolonged dry periods and under extreme heat [1]. Additionally, it contributes to soil stabilization and microclimate regulation, supporting biodiversity in otherwise inhospitable desert environments.

*Prosopis cineraria* holds substantial cultural and ethnobotanical importance within the region. Historically, its canopy has provided shelter for communal and ceremonial gatherings, while its foliage and seed pods have served as nutritional resources for both humans and domesticated animals. The species is widely regarded as a symbol of resilience, hospitality, and social cohesion, as reflected in the regional folklore and literary traditions.

Despite its ecological and cultural significance, *P. cineraria* faces increasing threats from urbanization, habitat loss, and overgrazing, particularly by camels. At the national level, it is currently assessed as ’Least Concern’ under the IUCN Red List criteria due to its relatively widespread distribution and persistence in protected areas [2]. However, within the Emirate of Abu Dhabi, local assessments and conservation efforts indicate that the species is considered endangered due to localized pressures and habitat fragmentation [3].

Given its dual role as a cultural symbol and a keystone of desert ecosystems, the conservation of *P. cineraria* requires approaches that extend beyond traditional ecological observations. While its ecological services and cultural relevance underscore its value, they also highlight the risks posed by unchecked habitat loss, overgrazing, and climate stress. Genomic data provide the critical layer needed to translate this cultural and ecological importance into practical conservation action by revealing patterns of genetic diversity, lineage distinctiveness, and adaptive potential. Establishing a genomic baseline is therefore essential to ensure that conservation strategies preserve not only the visible presence of *P. cineraria* in the landscape but also the hidden genetic resilience that supports its ecological and cultural legacy.

Despite its importance, *P. cineraria* has been largely understudied in global genomic initiatives, and little is known about its population-level genetic variation. This knowledge gap is particularly pressing given the escalating pressures of climate change, habitat fragmentation, and land-use change across arid regions. Understanding the genetic diversity and structure of *P. cineraria* is essential for evidence-based conservation, restoration, and management strategies [4].

Recent genomic studies in desert and arid-adapted trees illustrate how such approaches can reveal both demographic history and adaptive potential. For example, genome survey sequencing of *Acacia* in Kuwait provided critical insights into the genetic status of an endangered desert tree [5]. A chromosome-scale genome and population analysis of desert poplars uncovered adaptive gene family expansions related to aridity [6]. Comparative work in the *Neltuma* and *Strombocarpa* legumes has illuminated patterns of divergence and molecular evolution in dryland habitats [7]. More broadly, integrative reviews emphasize the importance of genomics for understanding desert plant physiology and adaptation [8]. Complementary efforts in arid crops, such as the recent chromosome-level genome assembly and annotation of *Citrullus colocynthis* [9], further demonstrate how genomic resources can accelerate research on resilience to drought and salinity.

Advances in computational methods and next-generation sequencing have transformed conservation genetics, enabling the analysis of hundreds of thousands of markers to uncover biodiversity traits, disease resistance, migration patterns, and adaptation. Whole-genome sequencing adds even deeper insight—although it requires rigorous data filtering and visualization to ensure reliability. Together, these tools sharpen our understanding of wild populations and their environments, powering population genomics studies that drive molecular ecology, evolutionary biology, and biodiversity conservation. This study was further empowered by the genome assembly of *Prosopis cineraria*, which laid the foundation for deeper genomic insights into this keystone desert species [10].

Here, we present the first population-scale genomic analysis of *P. cineraria* based on 204 individuals sampled across the UAE. Leveraging whole-genome resequencing and SNP-based analyses, our aims are to (1) characterize the population structure and patterns of genetic diversity across its range, (2) identify genomic regions under differentiation that may signal local adaptation, and (3) investigate gene flow and historical connectivity among groups. This work, carried out from the partnership of the International Center for Biosaline Agriculture (ICBA) and the Environment Agency–Abu Dhabi (EAD), provides a genomic baseline to support evidence-based conservation, restoration, and genetic monitoring of this culturally and ecologically significant species.

Our findings are framed not only by evolutionary and ecological questions but also by national conservation goals, supporting seed sourcing programs, restoration ecology, and long-term genetic monitoring. Importantly, this research can help to identify and prioritize high-value populations for ex situ conservation efforts, including gene banking and seed banking, thereby safeguarding genetic diversity for future restoration and resilience initiatives.

By generating the first genome-scale dataset for this iconic desert species, our study not only establishes a critical foundation for conservation genomics in the Arabian Peninsula but also delivers practical tools, such as interactive genomic maps, that can directly support conservation planning, restoration programs, and long-term biodiversity stewardship.

## 2. Materials and Methods

A summarized visual scheme of the experimental workflow from sample collection to bioinformatics analyses is shown in Supplementary Figure S1.

### 2.1. Sample Collection, SNP Calling, and Filtering

A total of 211 *Prosopis cineraria* individuals were collected across multiple locations in the United Arab Emirates based on the species’ distribution across major environmental and geographic zones, including open deserts, mountainous regions, alluvial plain, and coastal areas.

The identification of *Prosopis cineraria* tree was carried out using a combination of comprehensive flora reference books, detailed monographic descriptions, and confirmation through comparison with herbarium specimens from the EAD herbarium collection. These resources allow for accurate identification in natural habitats by examining distinctive features, including foliage, bark texture, and overall morphology.

Sampling aimed to capture the full geographic and ecological range of *Prosopis cineraria* in the UAE. The collecting areas and routes to be followed were determined according to the geographical areas of the UAE, the coastal lowlands in the west and east, the western dune plains, the central desert, the alluvial plain, and the mountain belt.

In this phase of collecting, eight expeditions were launched, covering most parts of the UAE. The sampling strategy depended on the population size and variation in habitat diversity. In general, for widely distributed species, stratified random sampling was adopted, and not less than 10 trees were sampled from each collecting site. For the ecotypes that were very rare and found in very small numbers, all the plants available at the site were sampled. In the absence of any morphological variation, samples of an accession collected from similar ecological habitats within each geographic area were pooled into one irrespective of the distance between the collecting sites. After being collected into plastic containers, the leaf samples were transferred immediately to liquid nitrogen for preservation and later for DNA extraction.

Upon identification, the collection of *Prosopis cineraria* tree leaves followed a structured protocol to ensure sample integrity. Leaves were carefully plucked using sterilized gloves to avoid contamination, and each leaf sample was placed into 50 mL Falcon tubes, which were clearly labeled with a unique identifier to maintain traceability. The exact location of each tree was recorded using GPS devices and registered in the EAD Geographic Information System (GIS) database, ensuring traceability of the samples and contributing to ecological studies and conservation efforts.

Following collection, the preservation and preparation of the *Prosopis cineraria* tree samples for analysis involved several steps. Upon collection, the Falcon tubes containing the samples were immediately stored in a dry ice box to prevent the degradation of biological material and preserve the samples’ viability for subsequent analysis. The samples were then transported under controlled conditions to ICBA’s Desert Life Science Laboratory (DLSL), where the preservation process continued. At the laboratory, the samples went through various preparatory procedures, including washing, drying, and grinding, to extract the necessary compounds for analysis. These steps were followed by standardized protocols to ensure consistency and reliability of the results. This systematic methodology guaranteed the integrity of the *Prosopis cineraria* tree samples throughout the process, enabling accurate and reliable analysis.

DNA was extracted from flash-frozen leaf samples using a modified CTAB method [11]. The purity and quality of the isolated DNA were verified by agarose gel electrophoresis on 0.8% agarose gels, and the concentration was determined on a Qubit 4 fluorometer using Qubit Broad Range Assay Kit (Thermo Fisher Scientific Inc., Waltham, MA, USA). One microgram of genomic DNA was mechanically fragmented to an average size of 250 bp using the Covaris^®^ M220 Focused-ultrasonicator™ (Covaris, Woburn, MA, USA), and the size selection of fragmented DNA was conducted using MGIEasy DNAClean beads (MGI Tech, Shenzhen, China). A single-stranded circular DNA library was prepared using MGIEasy Universal DNA Library Prep Set Ver. 1.0 following the manufacturer’s standard protocol for a 250-base-pair insert size, followed by DNA nanoball (DNB) formation based on rolling circle amplification. The DNB was loaded into the flow cell (DNBSEQ-G400RS Sequencing Flow Cell Ver. 3.0), and cPAS-based 100-base-pair paired-end sequencing was performed with DNBSEQ-G400RS High-Throughput Sequencing Set Ver. 3.1 (MGI Tech).

Raw reads were demultiplexed and quality-checked using FastQC v0.11.9 [12]. Reads were trimmed and filtered using fastp v0.20.1 [13] and aligned to the *P. cineraria* reference genome using BWA-MEM v0.7.17 [14]. Duplicates were marked using Picard Tools v2.26.10 [15]. SNP calling was performed jointly across all samples using GATK HaplotypeCaller v4.5.0.0 [16] in GVCF mode, followed by joint genotyping with GenotypeGVCFs. The resulting raw VCF files were filtered with VCFtools v0.1.16 [17] by selecting biallelic SNPs only with minimum site quality of QUAL ≥ 30; minimum genotype quality GQ ≥ 20; minimum depth per genotype DP ≥ 5; maximum missingness per site < 10%; and minor allele frequency MAF ≥ 0.05.

Linkage disequilibrium (LD) pruning was then applied using PLINK v1.90b6.22 [18] with a sliding window of 50 SNPs, a step size of 5, and an $r^{2}$ threshold of 0.5. This yielded a final set of 57,183 high-quality LD-pruned SNPs.

Pairwise identity-by-descent (IBD) was computed with plink --genome. Sample pairs with PI_HAT ≥ 0.95 were considered clonemates; clonal groups were formed by transitive closure. Within each clonal group, we retained the sample with the lowest per-sample missingness (F_MISS) and excluded the others. Samples with >20% missing genotypes were excluded prior to analysis. A subset of 114 non-clonal samples was defined, and all downstream population structure and diversity analyses were performed both on the full dataset and the non-clonal subset depending on the objective.

### 2.2. Population Structure Analyses

To investigate the genetic structure within the *Prosopis cineraria* population, we employed sparse non-negative matrix factorization (sNMF) v1.2 [19]. Analyses were conducted using the LD-pruned SNP dataset of 57,183 SNPs from 114 individuals.

We tested values of K (number of ancestral populations) ranging from K = 1 to K = 10, with 20 replicates per K. The optimal K was selected based on the cross-entropy criterion, which minimizes prediction error.

Admixture coefficients (Q-matrix) for each individual were visualized as stacked barplots and integrated with geographic data using pie charts plotted on a map. To address sample overlaps at identical or near-identical GPS coordinates, we developed a flower expansion algorithm that dynamically separates overlapping pies, implemented in a custom interactive web app [20]. Clusters were defined based on sNMF results at the optimal K = 4. For interpretation, individuals were assigned to the cluster corresponding to their highest ancestry proportion.

In addition, individual assignments were compared to discriminant analysis of principal components (DAPC) that was conducted using the adegenet v2.1.11 [21] package. Input genotypes were converted to a genlight object, and population clusters (K = 4) were defined based on sNMF assignment. DAPC was performed using 40 principal components and 3 discriminant functions. Posterior membership probabilities and discriminant loadings were extracted for comparison with sNMF results.

### 2.3. Genetic Diversity and Differentiation

To assess genetic diversity within and between groups, we calculated standard population genetic statistics using the LD-pruned non-clonal dataset (114 individuals and 57,183 SNPs).

Observed heterozygosity (Ho) and expected heterozygosity (He) were computed using PLINK v1.9 and custom R scripts. The inbreeding coefficient (F) was calculated as $1-\frac{H_{o}}{H_{e}}$. A proxy for per-sample nucleotide diversity (π) was also estimated based on the observed heterozygosity, normalized by total loci.

To evaluate group-level differences in these diversity indices (Ho, He, F, and π), we applied ANOVA followed by Tukey’s HSD for post hoc comparisons. Visualizations included violin plots, overlaid scatterplots, and summary tables, all produced in R v4.3.3 [22] and Python v3.10.1 [23] custom scripts.

Genes overlapping the top 1% $F_{ST}$ windows were identified using the *P. cineraria* reference GFF3 annotation [10] and submitted for functional enrichment analysis using eggNOG-mapper v2.1.12 [24] on the v5.0.2 eggNOG database. Enrichment was conducted for Gene Ontology (GO) terms and KEGG pathways, with additional visualization using GOATOOLS v1.4.12 [25].

### 2.4. Functional Divergence and Annotation

To estimate genetic differentiation between groups, we calculated pairwise Weir and Cockerham’s fixation index ($F_{ST}$) using VCFtools v0.1.16, applied both genome-wide and in sliding windows of 50 kb and a 10 kb step, to scan for regions of elevated differentiation.

Windows falling in the top 1% of the genome-wide $F_{ST}$ distribution were considered candidate regions of divergence. These windows were intersected with the *P. cineraria* genome annotation in GFF3 format to identify overlapping or nearby genes.

Genes overlapping high-$F_{ST}$ windows were extracted and annotated using eggNOG-mapper v2.1.12, referencing the eggNOG v5.0.2 database. Functional annotation included Gene Ontology [26] terms (biological process, molecular function, and cellular component); KEGG orthology [27] and pathway assignments; and Pfam [28], InterPro [29], and UniProt [30] functional domains.

GO term enrichment analysis was conducted using GOATOOLS with a background set of all annotated genes in the genome. Enriched GO categories were filtered at FDR < 0.05. KEGG pathways were summarized using KO-term abundance mapping to the ko00001.keg hierarchy, and top pathways were visualized with matplotlib v3.10.1 [31].

### 2.5. Phylogenetic Analysis

To visualize genetic relationships among *Prosopis cineraria* individuals, we constructed a neighbor-joining (NJ) phylogenetic tree based on pairwise genetic distances.

Distances were calculated from the LD-pruned SNP dataset with fastreeR v2.0.0 [32] that uses cosine genetic distance as the distance metric.

To assess node support, we performed 1000 bootstrap replicates by resampling SNPs with replacement and generated consensus support values for each node. The resulting bootstrap values were mapped onto the NJ topology, and NJ tree was exported in Newick format; visualized with the iTOL v6 [33] online tool and a custom-built interactive web-based tree viewer [20]; and colored by sNMF group assignments for comparison with admixture results.

### 2.6. Multivariate and Clustering Analysis (DAPC)

To complement the sNMF population structure analysis, we performed discriminant analysis of principal components (DAPC) using the adegenet package in R.

We used the LD-pruned non-clonal genotype dataset (57,183 SNPs; 114 individuals) and followed this pipeline: SNP genotypes were imported and converted into a genlight object; group assignments were defined based on sNMF clustering at K = 4; principal component analysis (PCA) was first applied for dimensionality reduction; the number of PCs (n.pca = 40) was chosen based on a-score optimization; and discriminant analysis retained 3 discriminant functions to separate the four clusters.

Posterior probabilities of group membership and SNP loadings were extracted for interpretation and cross-validation against sNMF results.

### 2.7. Gene Flow and Admixture

We explored gene flow between the inferred *Prosopis cineraria* groups using two complementary approaches:

TreeMix Analysis: we used TreeMix v1.13 [34] to infer population splits and migration events; the LD-pruned non-clonal SNP dataset (57,183 SNPs; 114 individuals) was converted to TreeMix input using PLINK and custom scripts; groups were defined using sNMF (K = 4), and allele frequency matrices were generated per group; we tested migration edges (m) from 0 to 5 and rooted the tree at either Group 2 or Group 4; tree topology, edge weights, and residual fit were visualized using custom Python v3.10 and R v4.4 scripts; and arrows indicate direction and strength of gene flow events.

ABBA–BABA: we used Dsuite v0.5r58 [35] to perform D-statistic (ABBA–BABA) tests on the unpruned 204-sample VCF; a population assignment file mapped samples to groups and specified outgroup individuals; both global and per-chromosome tests were conducted, with the statistics D, Z-score, *p*-value, f_4-ratio, and site patterns (BBAA, ABBA, and BABA) computed; and per-chromosome results highlighted local introgression events.

### 2.8. Linkage Disequilibrium Decay

We evaluated linkage disequilibrium (LD) decay across the genome and among groups using PopLDdecay v3.43 [36]: LD was calculated on the LD-pruned non-clonal dataset (114 samples; 57,183 SNPs); analyses were performed per chromosome (PC1–PC15), per sNMF group (Group 1 to Group 4), or combined groups (ALL); and output files containing pairwise $r^{2}$ values and distances were generated for each (group to chromosome) combination.

For visualization: a custom Python pipeline parsed the stat files, binned $r^{2}$ by physical distance, and fit generalized additive models (GAMs) using the pygam v0.9.1 [37] library; LD decay curves were plotted for all groups together across all chromosomes and per chromosome (optionally selecting subsets of groups); and, for each curve, we calculated the half-decay distance (bp) at which $r^{2}$ dropped to 50% of its maximum.

## 3. Results

### 3.1. SNP Filtering and Dataset Summary

Out of the 211 initially sequenced *P. cineraria* samples, 204 passed quality filtering and were retained for downstream analyses. Seven samples with excessive missing data (more than 20% missing genotype calls) and hence low coverage were excluded.

**Figure 1:**
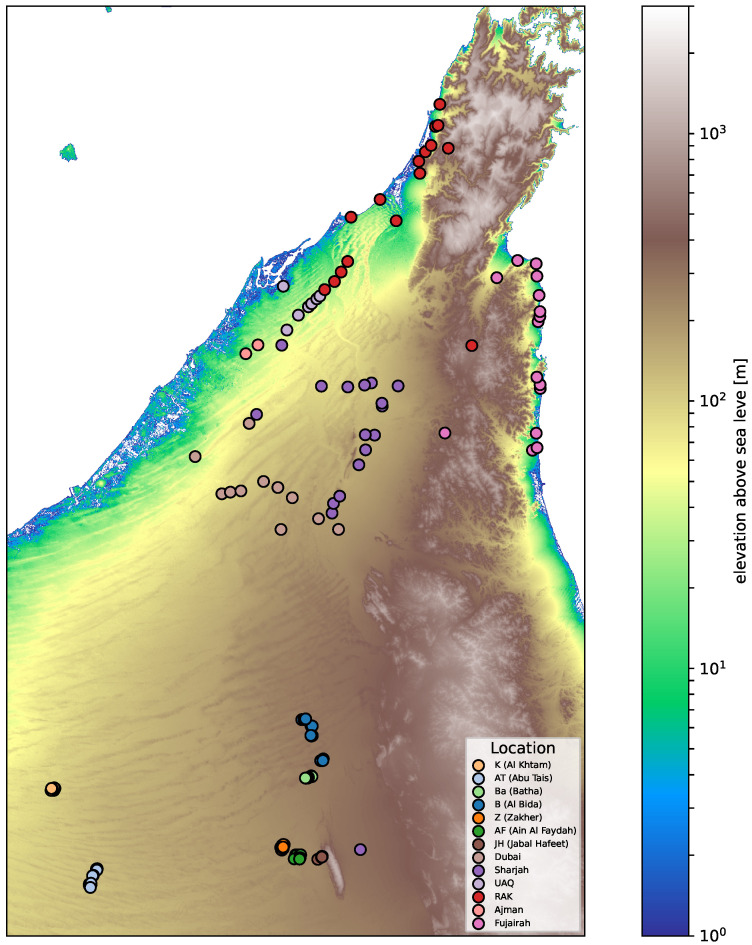
Sampling locations of *Prosopis cineraria* across the United Arab Emirates (UAE). The map shows 204 sampled trees over a shaded-relief basemap. Points indicate sample coordinates, color-coded by site. Sampling spans coastal and alluvial plains, inland sand-dune fields, and the Hajar foothills, providing geographic context for population analyses.

Geographic coordinates were available for all the retained samples, allowing integration with spatial analyses (Figure 1). Of the 204 individuals, 114 were genetically unique (non-clonal) and 90 belonged to clonal groups identified at PI_HAT ≥ 0.95. Representative samples were selected per group based on lowest missingness for downstream analyses The subset of 114 non-clonal samples was used in downstream population genetic analyses requiring unrelated individuals.

The whole-genome resequencing of 204 *Prosopis cineraria* individuals yielded a total of 37,302,514 raw SNPs. After applying quality and filtering criteria to retain high-confidence biallelic variants, 432,753 SNPs remained. Linkage disequilibrium (LD) pruning further reduced the dataset to 57,183 SNPs, which formed the basis for all the downstream population genomic analyses (Table 1).

### 3.2. Population Structure

**Figure 2:**
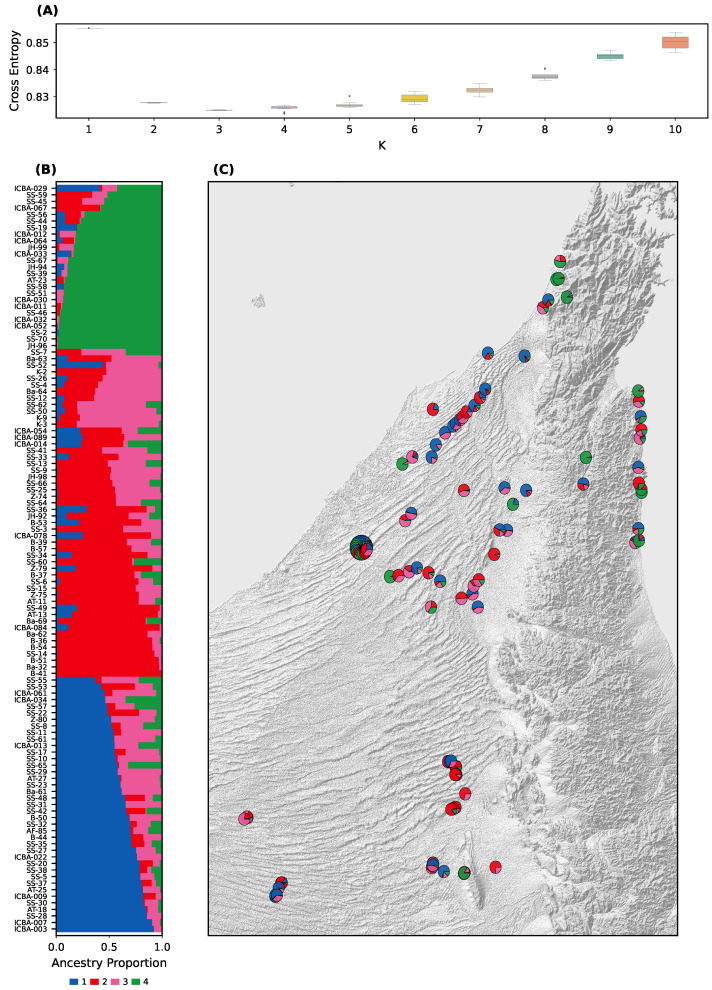
Population structure of *Prosopis cineraria* based on 57,183 LD-pruned SNPs from 114 non-clonal individuals. (**A**) Cross-entropy criterion for K = 1–10 (*X*-axis: K values; *Y*-axis: cross-entropy), with optimal clustering at K = 4. (**B**) Q-matrix barplot showing individual ancestry proportions (*X*-axis: individuals; *Y*-axis: ancestry proportion), sorted and grouped by major cluster. (**C**) Geographic distribution of ancestry proportions for the four genetic clusters inferred with sNMF. Each pie chart represents one sample, with colors corresponding to the four groups. Group 4 is concentrated in the northern and northeastern sites, whereas the other clusters are broadly distributed across the UAE.

A population structure analysis using sNMF revealed the presence of four major genetic clusters (K = 4). The optimal K was determined based on the minimum cross-entropy value, which sharply plateaued at K = 4 (Figure 2A).

sNMF proportions showed variable levels of ancestral mixing across individuals, with some genetically homogeneous clusters and others showing significant admixture. Notably, Group 1 and Group 3 shared substantial ancestry; and Group 2 and Group 4 also showed affinity, with Group 4 displaying the most genetic differentiation.

sNMF patterns were visualized through stacked barplots (Figure 2B), spatial pie charts, and geographic maps (Figure 2C). A custom web-based visualization tool [20] was developed to enable interactive exploration of the data, including geolocation and sNMF pie charts with overlapping samples resolved via flower-style expansions.

Among the four sNMF clusters, only Group 4 showed a clear geographic signal, being concentrated in the northern and northeastern sites (Figure 2C). The other groups were more broadly distributed across the UAE without distinct geographic boundaries. This pattern indicates that strong geographic barriers are absent for most clusters, and genetic connectivity likely reflects both natural dispersal and human-mediated planting.

**Figure 3:**
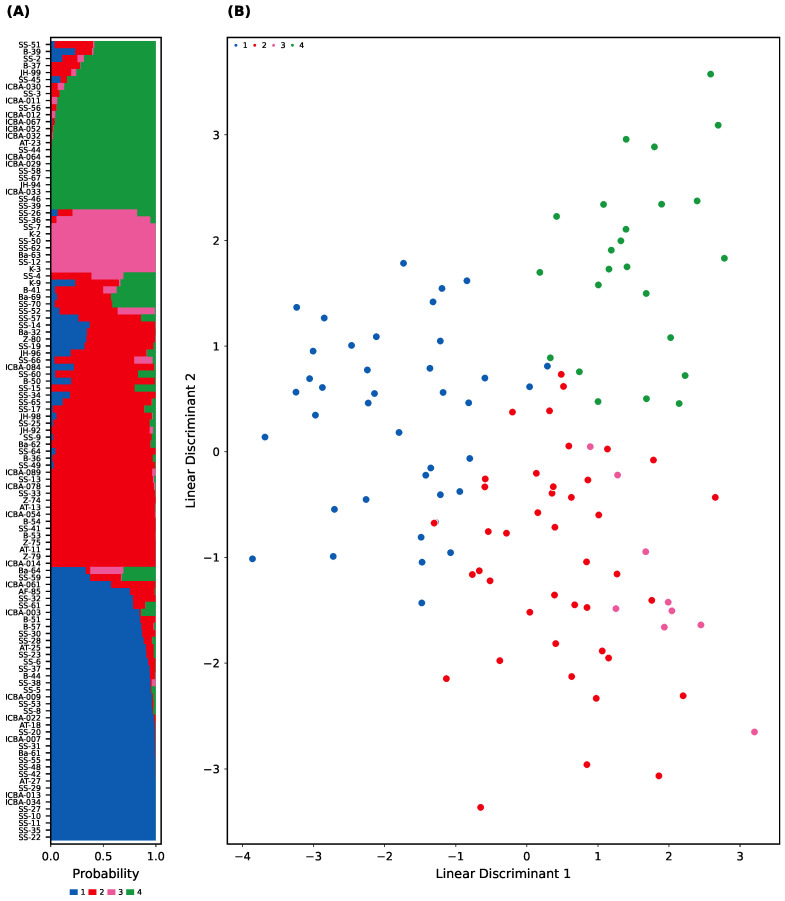
Discriminant analysis of principal components (DAPC) based on sNMF-defined groups (K = 4). The analysis confirms four genetically distinct clusters with high membership probabilities, closely mirroring the sNMF results. (**A**) Posterior membership probabilities of samples across groups (*X*-axis: individuals; *Y*-axis: membership probability). (**B**) Scatterplot of the first two linear discriminants (*X*-axis: LD1; *Y*-axis: LD2), with individuals colored by inferred group.

To further validate this structure, DAPC was applied to the same SNP dataset using sNMF-based groupings. The DAPC successfully discriminated the four clusters using just three discriminant functions (Figure 3A). The posterior membership probabilities were high for most individuals (≥90%), with a small subset of samples showing admixed or ambiguous ancestry (Figure 3B).

We also compared and confirmed individual assignment concordance between sNMF and DAPC using an alluvial plot (Supplementary Figure S2).

### 3.3. Genetic Diversity and Differentiation

**Figure 4:**
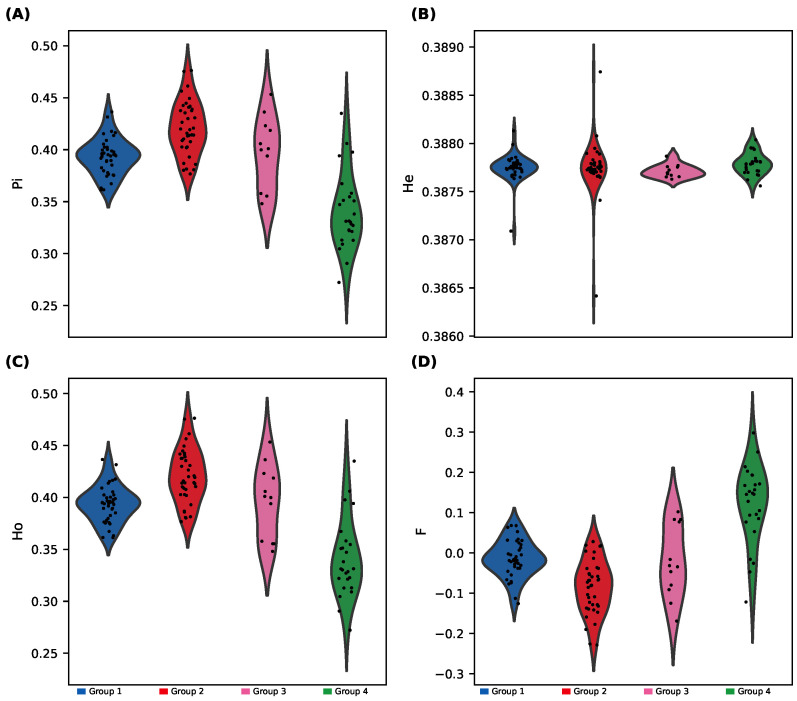
Genetic diversity indices across sNMF-defined groups (114 non-clonal individuals; 57,183 SNPs). Violin plots with scatter overlays show four diversity indices. Group 4 exhibits lower diversity and higher inbreeding. Group differences were tested with ANOVA. (**A**) Nucleotide diversity (π). (**B**) Expected heterozygosity ($H_{e}$). (**C**) Observed heterozygosity ($H_{o}$). (**D**) Inbreeding coefficient (F). (*X*-axis: groups; *Y*-axis: diversity index value).

We observed substantial differences in genetic diversity among the four sNMF-inferred groups (Figure 4; Supplementary Figure S3): Group 4 showed significantly lower heterozygosity (Ho and He) and nucleotide diversity (π), along with higher inbreeding coefficient (F); and Group 1 and Group 3 displayed the highest diversity metrics, while Group 2 was intermediate.

ANOVA tests followed by Tukey’s HSD confirmed that Group 4 differed significantly from the other groups for most diversity metrics.

**Figure 5:**
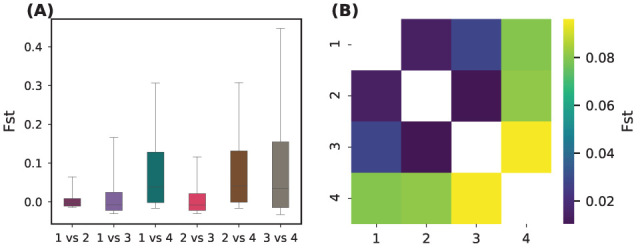
Pairwise genetic differentiation among sNMF-defined groups. Genetic differentiation is strongest between Group 4 and the other groups, particularly Group 3 ($F_{\mathrm{ST}}$ = 0.113), whereas Groups 1 and 2 show low differentiation. (**A**) Boxplots of SNP-wise $F_{\mathrm{ST}}$ values for all six group comparisons (*X*-axis: group pairs; *Y*-axis: $F_{\mathrm{ST}}$). (**B**) Symmetric heatmap of mean pairwise $F_{\mathrm{ST}}$ values (color scale: mean $F_{\mathrm{ST}}$).

**Figure 6:**
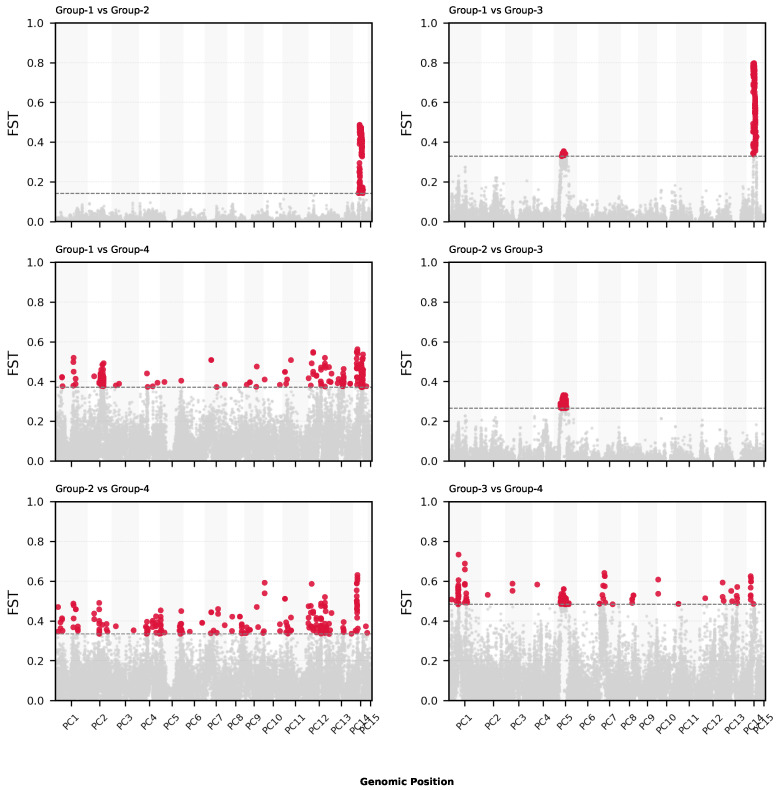
Genome-wide windowed $F_{\mathrm{ST}}$ across pairwise group comparisons. Sliding-window scans (50 kb windows, 10 kb step) highlight candidate divergent regions. The figure shows Manhattan-style plots for all six pairwise comparisons (*X*-axis: genomic position; *Y*-axis: windowed $F_{\mathrm{ST}}$). Points above the 99th percentile threshold (dashed line) are marked as outliers. These outlier regions were intersected with gene models for downstream functional analysis.

Pairwise $F_{ST}$ comparisons indicated moderate to strong differentiation, particularly involving Group 4 (Table 2; Figure 5):

We also performed a genome-wide $F_{\mathrm{ST}}$ scan in sliding windows (50 kb, step 10 kb) to identify genomic regions under potential differentiation (Figure 6; Supplementary Figure S4).

These results confirm that Group 4 is genetically distinct, with lower intra-group diversity and higher divergence from the rest.

### 3.4. Functional Divergence and Enrichment Analysis

To link regions of high genetic differentiation to potential functional relevance, we extracted the top 1% $F_{\mathrm{ST}}$ windows (±2500 bp) and intersected them with gene models (Figure 6).

**Figure 7:**
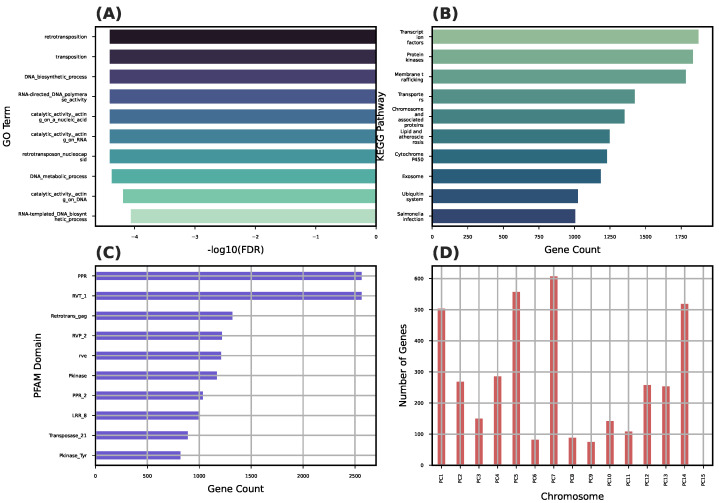
Functional enrichment of high-$F_{\mathrm{ST}}$ genes. Genes in highly divergent regions show enrichment for stress response, signaling, and metabolic functions. Divergent regions overlap with kinase domains, transporters, and retrotransposon activity. (**A**) Top enriched Gene Ontology (GO) terms (FDR < 0.05; *X*-axis: GO categories; *Y*-axis: enrichment score). (**B**) KEGG pathway enrichment (*X*-axis: pathways; *Y*-axis: enrichment score). (**C**) Most frequent Pfam domains among high-$F_{\mathrm{ST}}$ genes (*X*-axis: Pfam domains; *Y*-axis: frequency). (**D**) Top divergent genes by $F_{\mathrm{ST}}$ per chromosome (*X*-axis: chromosomes; *Y*-axis: top-ranked genes).

A total of 3960 genes were found to contain or overlap high-$F_{\mathrm{ST}}$ SNPs (Table 3). These genes were annotated using eggNOG-mapper (GO, KEGG, and PFAM). The enrichment analysis (FDR < 0.05) showed functional enrichment in processes related to stress response, kinase activity, membrane localization, and secondary metabolism (Figure 7).

Among these genes, ∼85% received functional annotation through eggNOG-mapper, 780 genes were assigned one or more GO terms, and 326 genes mapped to KEGG orthologs (KOs), representing 118 distinct KEGG pathways.

The Gene Ontology (GO) enrichment analysis revealed several overrepresented biological processes, including response to stress, signal transduction, and protein phosphorylation. At the molecular function level, the enriched terms included kinase activity, ATP binding, and metal ion binding.

The KEGG pathway analysis identified several significantly enriched and high-abundance pathways, notably plant hormone signal transduction, MAPK signaling, biosynthesis of secondary metabolites, and pathways associated with environmental adaptation.

These findings suggest that genes under high differentiation among *Prosopis cineraria* genetic clusters are involved in stress response, signaling, and local adaptation processes, consistent with ecological divergence in the UAE desert environment.

### 3.5. Phylogenetic Analysis

**Figure 8:**
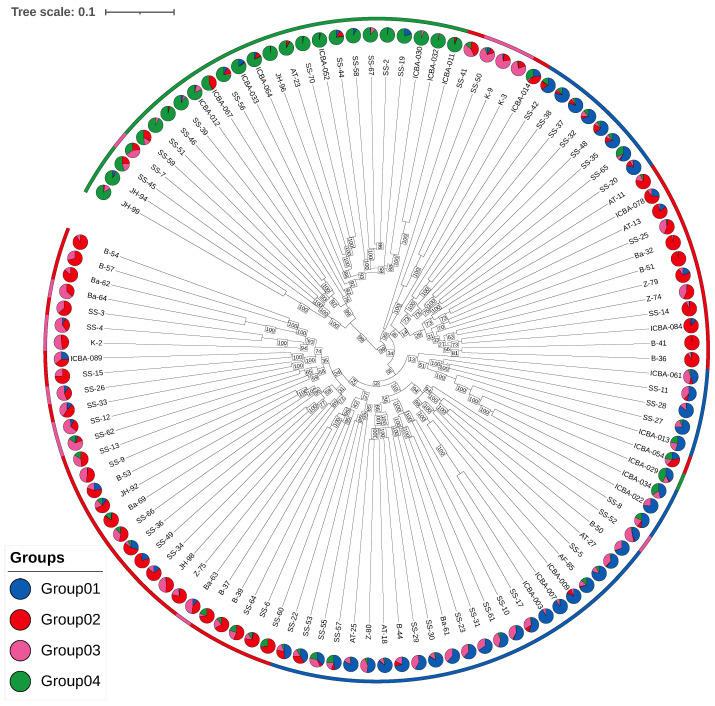
Phylogenetic relationships among *Prosopis cineraria* individuals with bootstrap support. A neighbor-joining tree was constructed from 57,183 LD-pruned SNPs (114 non-clonal individuals). Branches are colored by sNMF group assignment, and bootstrap support values (1000 SNP resampling replicates) are shown at major nodes. High support (>90%) corroborates the four main genetic clusters, with Group 4 forming a well-supported distinct lineage.

The neighbor-joining (NJ) tree (Figure 8) constructed from 57,183 LD-pruned SNPs revealed clear clustering of individuals consistent with the four sNMF groups. Group 1 and Group 3 formed a cohesive clade, indicating close genetic proximity. In contrast, Group 2 and Group 4 clustered separately, with Group 4 forming a more distinct and more divergent branch, consistent with its elevated $F_{ST}$ and higher inbreeding coefficients.

The bootstrap support values (1000 replicates) were high for all the major nodes, particularly the split separating Group 4 from the remaining lineages (>95%). This confirms the robustness of the clustering and supports the distinctiveness of Group 4.

The phylogenetic structure corroborates both the sNMF and DAPC clustering results, further supporting the presence of four distinct genetic lineages within the UAE *P. cineraria* population.

An interactive web-based tree viewer [20] allows dynamic exploration of the tree and access to individual sample metadata.

### 3.6. Gene Flow and Admixture

#### 3.6.1. TreeMix Results

Population splits and migration edges were inferred using TreeMix across the four sNMF-defined distinct genetic lineages within the UAE *P. cineraria* population (Supplementary Figure S5). All the tested models converged on similar topologies: Group 1 and Group 3 clustered together, while Group 2 and Group 4 formed a separate branch. Notably, a single migration edge consistently emerged between Group 1 and Group 2, suggesting historical gene flow between them.

#### 3.6.2. ABBA–BABA (Dsuite)

The genome-wide analysis using the ABBA–BABA (D-statistic) test revealed no significant global signal of introgression, indicating limited overall gene flow. However, chromosome-level tests uncovered localized signatures of gene flow, particularly on chromosomes PC1, PC5, PC8, PC9, PC12, and PC14. In several of these chromosomes, the Z-scores exceeded 2, supporting hypotheses of introgression at specific loci (Supplementary Figure S6). These results provide independent evidence for localized gene flow despite the overall well-differentiated population structure.

### 3.7. Linkage Disequilibrium Decay

**Figure 9:**
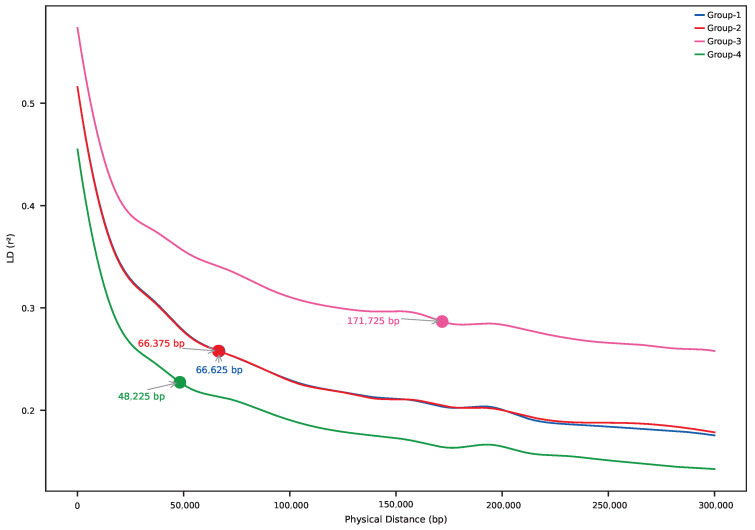
Linkage disequilibrium (LD) decay profiles across groups (114 non-clonal individuals; 57,183 SNPs). Group 4 shows steeper LD decay, whereas Group 3 displays a shallower profile. (*X*-axis: physical distance between SNPs; *Y*-axis: *r*^2^, the squared correlation coefficient). Steeper decay (as in Group 4) generally reflects larger long-term effective population size (*N_e_*) and/or higher effective recombination, while shallower decay (Groups 1 and 3) can result from admixture, structure, or smaller long-term *N_e_*.

The LD decay patterns varied among the groups and across the chromosomes. Group 4 consistently exhibited steeper decay curves and shorter half-decay distances, a pattern that is more consistent with a larger long-term effective population size and/or higher effective recombination than with a recent bottleneck (Figure 9). In contrast, Group 1 and Group 3 displayed shallower LD decay, which can arise from admixture/structure or smaller long-term *N_e_*. The chromosome-level LD decay plots further highlighted genomic heterogeneity, with chromosomes such as PC1, PC5, PC8, and PC14 showing evidence of longer-range LD (Supplementary Figure S7).

## 4. Discussion

Our study presents the first population-scale genomic assessment of *Prosopis cineraria* (Ghaf tree) in the United Arab Emirates, leveraging genome-wide SNPs, spatial coordinates, and functional annotations. The findings illuminate how geography, genetic drift, and localized selection have shaped the species’ genomic profile across its arid habitat.

One limitation of our study is that the results are based on a set of quality-filtered and LD-pruned SNPs. While such filtering is standard in population genomics to minimize false positives and linkage artifacts, it may also remove rare alleles and reduce the representation of long-range LD patterns. As a result, estimates of diversity and differentiation may be slightly conservative. Nonetheless, these steps enhance the reliability of the dataset, and the main signals of structure, differentiation, and gene flow are unlikely to be artifacts of the filtering procedure.

### 4.1. Four Genetic Clusters Reflect Geography and Admixture

We identified four well-supported genetic clusters using sNMF and DAPC (Figure 2 and Figure 3), consistent with subtle isolation-by-distance and regionally distinct demes. Groups 1 and 3 and Groups 2 and 4 formed two broader genetic meta-clusters with partial admixture, likely reflecting historical connectivity and subsequent drift.

Groups 1, 2, and 3 are closely related, with $F_{\mathrm{ST}}<0.04$ between any pair, consistent with low-to-moderate structure and potential gene flow or recent divergence. Group 4 is clearly distinct: it shows moderate-to-high differentiation from all the others ($F_{\mathrm{ST}}>0.09$). The highest value of $F_{\mathrm{ST}},\;0.1129$, against Group 3 indicated relatively strong divergence. The progression of $F_{\mathrm{ST}}$ values suggests that Group 4 may represent a geographically or ecologically isolated lineage, possibly under distinct selective pressures or with reduced gene flow.

These patterns align with biogeographic expectations for a long-lived insect-pollinated desert tree in fragmented habitats [38]. The availability of GPS-linked samples enabled spatial interpretation of genetic clusters (Figure 2C), with localized structure and pockets of genetic homogeneity near protected or isolated sites.

### 4.2. Group 4: A Genetically Distinct and Vulnerable Lineage

Group 4 emerged as a genetically depauperate and differentiated cluster, showing lower heterozygosity, higher inbreeding coefficients (Figure 4), and strong pairwise $F_{\mathrm{ST}}$ values (Figure 5). This suggests isolation, drift, and potential bottlenecks, a warning signal for long-term viability if unmonitored [4].

It contains samples like SS–44, SS–45, SS–67, etc., which are located far northeast, in the Hajar Mountains, the coastal Ras Al Khaimah, and northern Fujairah region. Respectively, many ICBA–XXX samples in this group are also genetically isolated despite being collected at the same ICBA site, suggesting a unique lineage possibly due to clonal propagation or a distinct origin. This group may represent either an ancestral lineage preserved in the northeast or a human-mediated introduction. Its strong divergence suggests limited gene flow with the others.

While our genomic analyses reveal Group 4 as a distinct lineage, a fuller understanding of its uniqueness requires integration with other lines of evidence. Integrating detailed morphological studies with genetic research would greatly enhance our understanding of lineage differences. Systematic assessments of morphological variation could validate and complement genetic data while also revealing adaptive traits linked to environmental pressures or historical factors. Such an approach would provide a broader perspective on the diversity within *Prosopis cineraria* and clarify how observable characteristics correspond to genetic structure. Prioritizing this integration in future research will strengthen conservation and restoration strategies.

By combining genetic and morphological perspectives, future research can generate the comprehensive evidence base needed to design targeted conservation actions and ensure the long-term resilience of this vulnerable lineage.

### 4.3. Signals of Local Adaptation in Differentiated Genomic Regions

The sliding-window $F_{\mathrm{ST}}$ scans (Figure 6) revealed several highly differentiated regions between group pairs, particularly between Group 1 and Group 4. The enrichment analyses of the genes overlapping these high-$F_{\mathrm{ST}}$ windows uncovered biological functions potentially linked to desert adaptation, including stress response, kinase signaling, and secondary metabolite biosynthesis (Figure 7; Table 3).

The GO enrichment results strongly suggest that divergent genomic regions between *Prosopis cineraria* subpopulations are enriched in transposable element-related functions, especially retrotransposons and genes involved in reverse transcriptase activity, polymerases, and nucleic acid remodeling. This enrichment in retrotransposon activity may signal active genome reshaping in *Prosopis cineraria* tree populations. This could be linked to adaptation, stress responses, or epigenetic regulation, especially relevant in the harsh arid environments of the UAE. This might further indicate a mechanism for generating genetic diversity in otherwise clonally propagated populations.

These findings echo previous ecological and transcriptomic studies suggesting that *P. cineraria* has evolved specific physiological responses to water scarcity, heat, and salinity. The identification [39] of candidate genes and pathways expands our understanding of the molecular basis of resilience in desert trees.

### 4.4. Evidence of Asymmetric Gene Flow

The TreeMix analysis (Supplementary Figure S5) consistently inferred a migration edge from Group 1 to Group 2, a signal that was mirrored in ABBA–BABA tests showing elevated D-statistics in several chromosomes (Supplementary Figure S6). While the global gene flow signals were weak, the chromosome-specific results suggest historical episodes of introgression, possibly linked to shared environmental pressures or secondary contact zones.

Such localized introgression may reflect past hybrid zones or stepping-stone gene flow facilitated by intermittent connectivity during wetter climatic periods [40]. *Prosopis cineraria* may represent a remnant species from a significantly wetter climatic period in the distant past. Its continued presence in the region underscores its resilience in the face of progressive aridification over the past approximately 5000 years [41,42].

### 4.5. Contrasting LD Decay Profiles Reflect Population History

The LD decay analysis revealed notable differences across the groups (Figure 9; Supplementary Figure S7). Group 4 showed the most rapid decay, implying smaller LD blocks and aligning with larger long-term $N_{e}$ and/or higher effective recombination rather than with a bottleneck. By contrast, Groups 1 and 3 exhibited shallower decay, which is compatible with admixture-induced LD, population structure, or reduced long-term $N_{e}$. We note that LD summaries can also be influenced by differences in minor-allele-frequency spectra, modest sample-size differences, and recombination-rate heterogeneity; accordingly, we present these as hypotheses to be tested with complementary analyses (e.g., ROH/IBD profiles, demographic inference, and fine-scale recombination maps).

To reconcile these patterns, we note that LD decay primarily reflects long-term effective population size and recombination dynamics, while heterozygosity and inbreeding coefficients capture more recent demographic processes. The steeper LD decay in Group 4, despite its lower diversity and higher inbreeding, may therefore arise from differences in allele frequency spectra, reduced admixture relative to other groups, or historically larger $N_{e}$, even if recent isolation has increased inbreeding. Future demographic modeling will be required to disentangle these processes.

As a precaution against interpretational bias, we avoid causal claims based solely on LD and will evaluate these alternatives in future work integrating ROH, IBD, and demographic modeling.

### 4.6. Implications for Conservation

In the case of *Myricaria laxiflora*, an endangered riparian shrub native to China’s Yangtze River Basin, the conservation of the *M. laxiflora* was heavily based on ex situ conservation. Reviewing the genetic diversity revealed a spatially biased sampling that overlooked a critical upstream genetic cluster, a missed cluster group that held unique variations vital for the plant’s long-term resilience [43].

Looking beyond this case, global reviews of plant conservation efforts have highlighted that many ex situ collections fail to adequately represent the genetic diversity found in wild populations [44]. This points to a systemic flaw in how ex situ conservation is often approached, with the risk of overlooking the full spectrum of genetic variation and thereby undermining the long-term goal of safeguarding species for future generations.

As the national tree of the United Arab Emirates, *Prosopis cineraria* holds ecological, cultural, and symbolic significance. It is recognized as a keystone species that is essential for desert ecosystems. It stabilizes sand dunes, enriches soil through nitrogen-fixing roots, and provides shade that supports microclimates. Its structure offers critical shelter and nesting for desert fauna, making it vital for biodiversity and desert restoration.

Our findings have direct implications for enhancing ecosystem resilience amid climate change. They support targeted conservation and restoration strategies that contribute to the development of climate-resilient landscapes across the arid habitat.

First, the four genetically distinct clusters identified through our analyses should be considered as separate conservation units. Notably, Group 4 appears to be particularly divergent and may require targeted in situ protection to avoid genetic swamping and preserve its unique lineage.

Second, the candidate genes and pathways identified as being under selection (Table 3 and Supplementary Data) highlight sources of adaptive genetic variation. These loci could be leveraged to guide assisted gene flow or targeted restoration, particularly in regions experiencing increasing desertification or salinity stress.

Finally, the development of interactive genomic maps and phylogenetic tree visualizers [20] (Figure 2C and Figure 8) provides practical tools for long-term monitoring and decision–making. These tools could support scalable implementation by UAE environmental authorities such as the Environment Agency Abu Dhabi (EAD).

A limitation of this study is that genetic variation was not directly compared with morphological or physiological traits, such as drought resistance. Such integrative analyses are important for translating genetic clusters into functional conservation units. Our results therefore provide a genomic baseline that can be combined with future ecological and morphological assessments to refine conservation and restoration strategies.

Altogether, our study illustrates how population genomic data can align with national policy objectives and contribute to evidence-based biodiversity stewardship in arid and semi-arid regions, particularly in biodiversity hotspots like the Arabian Peninsula, where conservation genomics remains an emerging discipline.

### 4.7. Future Directions

This study lays a strong foundation for continued genomic, ecological, and conservation research on *Prosopis cineraria*. Several avenues merit further exploration. High-coverage whole-genome resequencing of representative individuals would enable the detection of regulatory and non-coding variants potentially involved in local adaptation. Functional validation of candidate adaptive genes could be achieved through transcriptomic profiling under stress conditions. Additionally, defining seed zones and developing assisted gene flow strategies will be essential, particularly for genetically depauperate populations such as Group 4. Comparative genomic analyses with other *Prosopis* species, or even unrelated desert-adapted trees from the region, could shed light on convergent evolutionary responses to arid environments.

Future studies should aim to combine genomic data with detailed phenotypic and environmental information to enable genome-wide association studies (GWASs). Such analyses would help to identify loci linked to adaptive traits, including drought and salinity tolerance, and thus provide direct guidance for conservation and restoration programs [45].

Expanding the current framework to include *P. cineraria* populations from neighboring countries (e.g., Oman) would provide a broader biogeographic perspective. Finally, integrating genomic insights into citizen science initiatives and national planting campaigns would help to promote long-term resilience in reforested or restored habitats.

The immediate implication is the need for targeted conservation actions for the genetically unique and vulnerable Group 4 to preserve its lineage and enhance its resilience.

To interpret these findings into practical actions, we propose the following for effective collaboration with regional institutions:

1. Joint Monitoring Program: Establish a long-term genetic monitoring program in collaboration with local and international environmental bodies. This program would track changes in genetic diversity and population structure over time, providing early warning signs for populations at risk.
2. Integrated Plant Genetic Resource Conservation: Partner with local and regional institutes and entities to develop an integrated germplasm conservation strategy. This would involve expanding ex situ collections in gene banks and seed banks, with a particular focus on capturing the unique genetic diversity of Group 4 while also strengthening in situ conservation efforts.
3. Collaborative Restoration and Research: Initiate collaborative research projects with academic institutions and environmental agencies to validate the function of adaptive genes and develop scientifically guided restoration protocols. This includes establishing pilot projects for assisted gene flow aimed at enhancing the resilience of vulnerable populations without compromising local adaptation.

## 5. Conclusions

This study provides the first comprehensive genomic snapshot assessment of *Prosopis cineraria* populations across the United Arab Emirates. Our analyses revealed a clear population structure influenced by geographic separation and admixture, alongside the presence of a genetically distinct and less diverse cluster (Group 4), which may warrant targeted conservation efforts. We identified genomic regions with strong differentiation, many of which are enriched for functions related to environmental adaptation. Patterns of historical gene flow were detected at the chromosome level, and variations in the linkage disequilibrium decay among the groups reflected differences in demographic history and recombination dynamics.

Beyond its scientific contribution, this work offers critical insights for the evidence-based conservation of *P. cineraria*, the national tree of the UAE. It also expands the genomic toolkit available for studying desert-adapted trees, thereby supporting regional strategies for biodiversity management and climate resilience in arid ecosystems.

## Figures and Tables

**Table 1:** SNP filtering pipeline and resulting dataset sizes. Details of each filtering stage and final dataset sizes (SNPs and samples).

Step	SNPs Retained	Samples Retained
Raw SNPs from joint genotyping	432,753	204
Quality-filtered (biallelic, depth, GQ, missingness, MAF)	120,618	204
LD-pruned ($r^{2}<0.5$)	57,183	204
Final non-clonal subset	57,183	114

**Table 2:** Pairwise $F_{\mathrm{ST}}$ matrix. Numeric $F_{\mathrm{ST}}$ values between all group combinations.

Comparison	*F*_ST_	Differentiation Level
Group 1 vs. Group 2	0.0166	Very low
Group 1 vs. Group 3	0.0363	Low
Group 1 vs. Group 4	0.0901	Moderate
Group 2 vs. Group 3	0.0139	Very low
Group 2 vs. Group 4	0.0926	Moderate
Group 3 vs. Group 4	0.1129	Moderately high

**Table 3:** High-$F_{\mathrm{ST}}$ regions per group comparison, collectively overlapping 3960 genes. The number of SNPs found in the top 1% $F_{\mathrm{ST}}$ regions per group comparison. Collapsed number of genes found spanning these SNPs (±2500 bp).

Comparison	Outlier SNP Count	Threshold *F*_ST_	Outlier Gene Count
Group 1 vs. Group 2	4349	0.4756	235
Group 1 vs. Group 3	4566	0.7959	220
Group 1 vs. Group 4	4290	0.4780	1283
Group 2 vs. Group 3	4286	0.2728	422
Group 2 vs. Group 4	4290	0.4345	1796
Group 3 vs. Group 4	4276	0.5690	1844

## Data Availability

All raw whole-genome sequencing reads from the 204 *Prosopis cineraria* individuals have been deposited in the European Nucleotide Archive (ENA), part of the INSDC, under accession number PRJEB82449. These include 100-base-pair paired-end reads generated on the DNBSEQ-G400RS platform. Variant call format (VCF) files from joint SNP calling, filtered and LD-pruned genotype matrices, sample metadata (collection site coordinates, clonal status, and population assignments), and summary results from population structure, *F*_ST_, and LD decay analyses are archived in Zenodo under DOI: 10.5281/zenodo.15716843.

## Supplementary Materials

The following supporting information can be downloaded at https://www.mdpi.com/article/10.3390/plants14192970/s1, Figure S1: Summarized visual scheme of the experimental workflow from sample collection to bioinformatics analyses. Figure S2: Concordance between sNMF and DAPC groupings. Alluvial (Sankey) diagram illustrating the correspondence of individual group assignments between sNMF and DAPC clustering approaches. Flow widths represent the number of shared individuals. Most individuals show consistent group membership across both methods. Figure S3: Per-sample nucleotide diversity π. Violin plot depicting the distribution of per-individual nucleotide diversity π, color-coded by sNMF group. Highlights within-group and between-group genetic variation. Figure S4: Genome-wide $F_{ST}$ scan: Group 1 vs. Group 4. Manhattan plot showing genome-wide $F_{ST}$ values in sliding windows. The top 1% most differentiated regions are highlighted, indicating candidate loci for population divergence. Figure S5: TreeMix inference of gene flow events. Maximum likelihood population tree inferred using TreeMix, showing inferred migration edges among Ghaf genetic groups. A notable migration event is detected from Group 1 to Group 2. Figure S6: ABBA–BABA D-statistic test across chromosomes. Barplot displaying chromosome-level D-statistics. Significant introgression signals (Z>2) are observed for chromosomes PC1, PC5, PC8, PC9, PC12, and PC14, indicating localized gene flow. Figure S7: Linkage disequilibrium (LD) decay by group and chromosome. Grid of LD decay curves ($r_{2}$ vs. physical distance) for each sNMF group across all 15 chromosomes. Group 4 shows a more rapid decay pattern, consistent with elevated inbreeding and reduced diversity.
